# Clinical, genetic, and epidemiological survey of Polish children and adolescents with severe obesity: A study protocol of the Polish–German study project on severe early-onset obesity

**DOI:** 10.3389/fendo.2022.972174

**Published:** 2022-11-21

**Authors:** Magdalena Mierzwa, Mirosław Bik-Multanowski, Michael B. Ranke, Stephanie Brandt, Bertram Flehmig, Ewa Małecka-Tendera, Artur Mazur, Elżbieta Petriczko, Martin Wabitsch, Małgorzata Wójcik, Agnieszka Zachurzok

**Affiliations:** ^1^ Pediatric Endocrinology Ward, Independent Public Clinical Hospital No. 1, Medical University of Silesia in Katowice, Zabrze, Poland; ^2^ Department of Medical Genetics, Faculty of Medicine, Jagiellonian University Medical College, Cracow, Poland; ^3^ Children’s Hospital, University of Tübingen, Tübingen, Germany; ^4^ Center for Rare Endocrine Diseases, Division of Pediatric Endocrinology and Diabetes, Department of Pediatrics and Adolescent Medicine, Ulm, Germany; ^5^ Mediagnost GmbH, Reutlingen, Germany; ^6^ Department of Pediatrics and Pediatric Endocrinology, Medical University of Silesia, School of Medicine in Katowice, Katowice, Poland; ^7^ Department of Pediatrics, Pediatric Endocrinology and Diabetes, Medical Faculty, University of Rzeszów, Rzeszów, Poland; ^8^ Department of Pediatrics, Endocrinology, Diabetology, Metabolic Disorders and Cardiology of Developmental Age, Pomeranian Medical University, Szczecin, Poland; ^9^ Department of Pediatric and Adolescent Endocrinology, Pediatric Institute, Jagiellonian University Medical College, Cracow, Poland; ^10^ Department of Pediatrics, Faculty of Medical Sciences in Zabrze, Medical University of Silesia, Zabrze, Poland

**Keywords:** monogenic obesity, leptin, severe early onset obesity, children, adolescents

## Abstract

Severe early-onset obesity (SEOO) in children is a common feature of monogenic obesity. Nowadays, mutations in at least 50 genes are known to be related to monogenic obesity, and many others are tested. Part of them is involved in the leptin–proopiomelanocortin pathway. The aim of the project is to establish the Polish database of severely obese children and adolescents and to evaluate the prevalence of monogenic forms of obesity in this cohort, with a special focus on leptin–proopiomelanocortin pathway abnormalities. The secondary project aim is to identify new population-specific mutations in obesity-related genes in severely obese Polish children and adolescents. This is a prospective multi-center clinical study performed in four Polish centers. The estimated sample size is 500 patients aged 1–18 years, with severe obesity, hyperphagia, and food-seeking behaviors. In each patient, the medical history regarding the obesity duration in the patient and obesity and its complication existence in the family will be taken. Next, the questionnaire regarding the symptom characteristic of specific mutations, which we are going to test, will be performed. Hyperphagia will be assessed on the basis of age-specific questionnaires. The physical examination with anthropometric measurement, basic biochemical and hormonal tests, and leptin and biologically active leptin measurements will be performed. Finally, genetic analysis will be performed using next-generation sequencing with sequencing libraries prepared to include obesity-related genes. The genotyping findings will be confirmed with the use of classic sequencing (Sanger’s method). In the future, the pathogenicity of new mutations in obesity-related genes identified in our cohort is planned to be confirmed by functional testing *in vitro*. Nowadays, there are no data regarding the prevalence of severe obesity or monogenic obesity in Polish children. This project has the potential to improve understanding of obesity etiology and may contribute to implementing attribute mutation-specific treatment. Moreover, it may lead to a finding of new, population-specific mutations related to SEOO.

## 1 Introduction

The obesity epidemic has become an extremely important medical and socioeconomic issue in many countries, as excessive body weight is the leading cause of increased morbidity and mortality. The risk of obesity and its consequences in adulthood are even higher if the problem starts in childhood. In some countries, efforts have been undertaken to prevent and treat childhood obesity leading to the stabilization of the number of cases. However, the proportion of severely obese adults and children is still growing, and this group is at high risk of morbidity and mortality.

It is well known that the most common reason for excessive body weight is an imbalance between calorie input and output, and it is strongly related to lifestyle. Reduced daily physical activity with increased calorie consumption enhanced by polygenic background is the leading cause of obesity. However, there is a group of patients in whom a monogenic cause of obesity can be identified. This group is usually characterized by severe obesity with early onset, before the age of 6 years (severe early-onset obesity (SEOO)) (1). Kohlsdorf et al. revealed in a retrospective cohort study that patients with monogenic obesity due to leptin or leptin receptor deficiency showed an enormous increase in body mass index (BMI) during the first 2 years of life. Their BMI was >25 kg/m^2^ at the age of 2 years and >30 kg/m^2^ at the age of 5 years (2). These BMI values may be useful in distinguishing the monogenetic form of obesity from common obesity based on trajectories of BMI during early childhood. Nevertheless, in patients with some mutations, for example, in the melanocortin-4 receptor gene (*MC4R*), the BMI trajectories are similar to those of patients with simple obesity (3). Skinner et al. (4) showed that 7% of girls and 8.7% of boys in the USA suffer from severe obesity defined as a BMI above 120% of the 95th percentile. In a Spanish cohort, 2% of preschool children were severely obese (5). In subjects with SEOO, depending on the country, population studied, ethnicity, and the number of genes tested, the prevalence of monogenic obesity could vary between 3% and 10% of cases (6–8). Akinci et al. in a cohort of 105 SEOO children screened for 41 known-obesity-related genes found the genetic background in 10.4% of them (6). Slovenian national study has revealed that 1.4% of participants had known disease-causing heterozygous variants (DCVs) in the genes of the leptin–melanocortin signaling pathway and that 4.1% of participants were carriers of rare variants of unknown clinical significance (VUS) (9). Additionally, population-specific mutations in obesity-related genes exist. In a US cohort of African American and Latino children with SEOO, three out of eight discovered mutations in MC4R gene were new and not previously reported (7). A systematic review of the genetics of monogenic obesity in the 22 Arab countries in 13 studies has revealed carried 14 variants in five genes related to monogenic obesity; all of these variants were pathogenic, homozygous, and carried by members of consanguineous families (10). Unfortunately, there are no data about the prevalence of severe obesity in Polish children as well as about the prevalence of monogenic obesity in Polish SEOO children.

The aim of our project was to establish the Polish database of severely obese children and adolescents and to evaluate the prevalence of monogenic forms of obesity in this cohort, with a special focus on leptin–proopiomelanocortin pathway abnormalities. The secondary project aim was to identify new population-specific mutations in obesity-related genes in severely obese Polish children and adolescents. This is the first research assessing the prevalence of monogenic obesity in these parts of Europe in the population of such homogeneity. In Poland, there are a few minorities of different origins who migrated from other parts of the world (Middle East, North Africa, etc.), and the majority of Polish citizens are Caucasians with a low incidence of consanguinity.

## 2 Study objectives

1. To establish a Polish database of children with severe obesity
2. To characterize clinically and biochemically patients with severe obesity
3. To assess leptin and bio-active leptin in children with severe obesity
4. To evaluate the prevalence of monogenic obesity among Polish children with severe obesity, especially in those with early-onset obesity
5. To identify new mutations in obesity-related genes specific to the population of Polish children with severe obesity

## 3 Study design

This is a prospective multi-center clinical study performed in four Polish centers. The sample size targeted is 500 patients aged 1–18 years, with severe obesity of an early origin, hyperphagia, and food-seeking behaviors.

## 4 Materials

### 4.1 Patients

#### 4.1.1 Sample size

Five hundred children aged 1–18 years, with severe obesity of an early origin, will be recruited, using the following inclusion and exclusion criteria. The patients will be recruited from four Polish centers of pediatric endocrinology involved in childhood obesity management (Zabrze, Cracow, Rzeszów, and Szczecin) from inpatient and outpatient departments. The child population in Poland was estimated to be about 7,000,000 (11); for a 5% error threshold and a test power of 0.95, the appropriate sample size was determined at 384 participants. Funds earmarked for this national project are sufficient for conducting a study on about half a thousand children. During the last 2 years, a widespread promotion campaign for this project has been carried out, among both the medical community and patients in Poland. Qualification criteria for the project were presented at medical conferences organized by Polish medical societies. Broad-based information campaign was held in the main Polish mass media through media presentations (interviews and radio and television studios). Through this action, children from the entire area of Poland will be enrolled.

#### 4.1.2 Inclusion criteria

1. Age 1–18 years
2. The presence of severe obesity will be defined as a BMI > 25 kg/m^2^ in a child below the age of 2 years, a BMI > 30 kg/m^2^ in children aged 2–6 years, a BMI > 35 kg/m^2^ in children aged 6–14 years, and BMI > 40 kg/m^2^ in children aged >14 years (2)
3. Hyperphagia and food-seeking behaviors
4. Written informed consent of the patient’s parent/guardian and patient above the age of 13 years to participate in the study

#### 4.1.3 Exclusion criteria

1. Lack of written informed written consent from patients’ parent/guardian or patient above the age of 13 years
2. Secondary cause of obesity: previously diagnosed genetic syndrome coexisting with obesity, treatment with medicine with known effect on weight gain (glucocorticoids, valproic acid, risperidone, and others), Cushing’s syndrome, and other secondary causes of obesity (note: patients with other endocrine diseases, especially deficiencies, will not be excluded, as they could be the presentation of monogenic obesity)

## 5 Data collection

A single visit of the patient in the study center will be needed. In each patient, the study procedures mentioned below will be performed:

1. Taking the medical history of the patient and the patient’s family (**Appendix 1: Case Report Form (CRF)**) regarding the obesity duration in the patient and obesity and its complication existence in the family
2. Questionnaire regarding symptoms characteristic of the specific mutation (**Appendix 2 of CRF**)
3. Hyperphagia assessment using the Polish version of questionnaires:
a. Children’s Eating Behavior Questionnaire (CEBQ) by Wardle for children below the age of 8 years (**Appendix 3a of CRF**) (12)—answered by parent/guardian
b. Three-Factor Eating Questionnaire (TFEQ): for children above 8 years of age (**Appendix 3b of CRF**) (13)—answered by the patient.
In order to avoid bias, the questionnaires will be performed prior to the contact with an investigator.
Each patient or guardian will be asked to fulfill a standard 9-day diary of physical activity and a diary of the diet.
4. Physical examination with anthropometric measurements (**Appendix 4 of CRF**). Body weight will be measured to the nearest 0.1 kg on a calibrated balance beam scale, and body height was measured to the nearest 0.1 cm. Waist circumference will be measured at the level of the midpoint between the lowest rib and iliac crest, and head circumference will be measured with the band to the nearest 0.1 cm. In all children, the pubertal stage (development of breast, genitalia, and pubic hair) will be documented according to the classification by Tanner. Blood pressure will be measured with calibrated automatic blood pressure monitor with the cuff size appropriate to the arm size in children sitting for at least 15 min before the examination.
5. Biochemical and hormonal tests (**Appendix 4 of CRF**) which are usually performed on every patient with obesity during routine management of the subject (at the first visit at the outpatient/inpatient department and then usually repeated once a year). They are performed in each center by using commercial, widely available methods. Oral glucose tolerance test (OGTT) results will be available from obese children above the age of 10 years or younger in whom puberty has begun (14).
6. Leptin and biologically active leptin assessment (15). Additional fasting blood taking (2–3 ml of blood) will required to obtain 1 ml of serum. Measurements of total leptin and biologically active leptin will be performed in the central laboratory, using ELISA.
7. Genetic analysis—next-generation sequencing. Sequencing libraries prepared to include 11 target genes (*LEP*, *LEPR*, *MC4R*, *SIM1*, *KSR1*, *POMC*, *PCSK1*, *NTRK2*, *MRAP2*, *SH2B1*, and *BDNF*), in addition to further analysis of other, less frequent, obesity-related genes (*UCP1*, *UCP3*, *CARTPT*, *DYRK1B*, *NROB2*, *PCSK2*, *PPARG*, *PPP1R3A*, *PPARGC1A*, *CCK*, *SLC2A4*, *TUB*, *ADCY3*, *SREBF1*, *ADRB2*, *ADRB3*, *AGRP*, *MC3R*, *ENPP1*, *PPARGC1B*, *PYY*, *SDC3*, *ADIPOQ*, *NAMPT*, *CFD*, *RETN*, *NPY*, *ADD1*, *PTPN1*, *IRS-1*, *GHRL*, *NEGR1*, *GIPR*, *TMEM18*, *FTO*, and *SLC22A1*) will be performed. The full names of the 11 genes, their location, type of inheritance, and phenotype characteristics of mutation are presented in **Table 1**. The possible function and clinical phenotype of the obesity-related genes that we examined are presented in **Table 2**.

**Table 1:** Most common genes associated with severe early-onset obesity.

Gene name	Inheritance	Type of mutation	Chromosome Location	Additional symptoms
Melanocortin 4 receptor (MC4R)	AR AD	Homozygous or compound heterozygous Heterozygous	18q21.32	Somatomegaly, increased head circumference, hyperinsulinemia
Leptin (LEP)	AR	Homozygous or compound heterozygous	7q32.1	Hypogonadotropic hypogonadism, hypothyroidism, growth hormone deficiency, immune deficiency
Leptin receptor (LEPR)	AR	Homozygous or compound heterozygous	1p31.3	Hypogonadotropic hypogonadism, hypothyroidism, growth hormone deficiency, immune deficiency
Single-minded homolog 1 (SIM1)	AD	Heterozygous, chromosomal rearrangement	6q16.3	Autism, behavioral problems
Kinase suppressor of Ras 2 (KSR2)	AD	Heterozygous	12q24.22-q24.23	Insulin resistance, low heart rate, reduced basal metabolic rate
Proopiomelanocortin (POMC)	AR AD	Homozygous or compound heterozygous Heterozygous	2p23.3	ACTH/adrenal insufficiency, abnormal pigmentation
Protein convertase subtilisin/kexin 1 (PCSK1)	AR AD	Homozygous or compound heterozygous Heterozygous	5q15	Neonatal diarrhea, hypoglycemia, hypothyroidism, adrenal insufficiency, diabetes insipidus
Neurotrophic receptor tyrosine kinase 2 (NTRK2)	AD	Heterozygous	9q21.33	Developmental delay, hyperactivity
Melanocortin 2 receptor accessory protein 2 (MRAP2)	AD	Heterozygous	6q14.2	none
SH2B adaptor protein 1 (SH2B1)	AD	Heterozygous	16p11.2	Severe insulin resistance, behavioral difficulties, developmental delay
Brain-derived neurotrophic factor (BDNF)	AD	Heterozygous	11p14.1	Developmental delay, hyperactivity

Note. AR, autosomal recessive; AD, autosomal dominant; ACTH, adrenocorticotropic hormone.

**Table 2:** The possible function and clinical phenotype of the obesity-related genes.

Possible function	Gene	Clinical phenotype
Leptin–melanocortin pathway	*LEP, LEPR, MC4R, POMC, PCSK1, SH2B1, PCSK2, CARTPT, MRAP2, SIM1, BDNF, NPY, AGRP, MC3R, IRS-1, SDC3*	Given in **Table 1**
Thermogenesis	*UCP1, UCP3, UCP2, PPARG PPARGC1A*,	Obesity, type 2 diabetes
Glucose oxidation	*KSR2*	Hyperphagia in childhood, low heart rate, reduced basal metabolic rate severe insulin resistance
Fatty acid oxidation	*KSR2, PPARGC1B*	Severe insulin resistance
Glycogen synthesis	*PPP1R3A*	Severe insulin resistance, lipodystrophy, type 2 diabetes, carotid intima media thickness
Adipogenic differentiation	*DYRK1B, PPARG*	Abdominal obesity, metabolic syndrome
Mesolimbic pathway—regulates appetite, gastric emptying	*CCK, PYY, GHRL*	
Incretin regulation (insulin secretion, gastric motility, nutrient absorption, food intake)	*GIPR*	
Glucose transport	*SLC2A4*	Severe insulin resistance, type 2 diabetes
Regulation of catecholamine function (lipolysis, energy expenditure)	*ADRB2, ADRB3*	Type 2 diabetes, lower resting metabolic rate, abdominal obesity, insulin resistance
Regulation Insulin Receptor	*NAMPT, IRS-1*	
Regulation Complement System (regulation of insulin production, glucose uptake, and triglyceride synthesis)	*CFD*	
Adipokine genes	*ADIPOQ, LEP, LEPR, RETN*	
Renin-angiotensin-aldosterone system (RAAS)	*ADD1*	
Regulation of insulin and leptin signaling	*PTPN1*	
Unclear/pleiotropic regulation of obesity	*NEGR1, TMEM18, FTO, SLC22A1, NR0B2, ADCY3*	

Genetic testing needed an additional 1 ml of blood taking (EDTA). The biological samples will be immediately sent to the Department of Medical Genetics for centralized analyses. For assessment of the presence of small gene variants, 100 ng of genomic DNA of every sample will be enzymatically fragmented (as determined at Sure Select XT and XT Low Input Enzymatic Fragmentation Protocol, Agilent Technologies, Santa Clara, CA, USA) and used for exome library preparation according to the SureSelectXT HS Target Enrichment System for Illumina Paired-End Multiplexed Sequencing Library protocol (Agilent Technologies). Next, the libraries will be tagged, pooled, and prepared for sequencing. Finally, the sequencing of the libraries will be performed by an external genotyping service provider (Macrogen Europe, Netherlands) with the use of the NovaSeq 6000 genomic sequencer. The genotyping findings will be confirmed with the use of classic sequencing (Sanger’s method) in the Department of Medical Genetics.

The raw genotyping data will be processed with the use of the DRAGEN Enrichment software (Illumina, San Diego, CA, USA) in order to obtain a table of all genetic variants in every patient. However, the final data analysis was restricted only to the *a priori*-selected set of genes of interest.

All data from the medical history, physical examination, and biochemical tests will be used to characterize the study group clinically and biochemically. The assessment of the prevalence of metabolic disturbances (lipid and carbohydrate disorders), metabolic-associated fatty liver disease, arterial hypertension, and metabolic syndrome will be performed, and their correlation with BMI z-score, bio-impedance data, and leptin and bioactive leptin level will be performed. To assess the leptin SDS, the Blum et al. formula will be used (16). The eating disorders will be analyzed on the basis of CEBQ and TFEQ. In children with an established mutation in genes related to obesity, a detailed clinical and biochemical data analysis will be performed, and further, necessary biochemical and hormonal tests will be performed.

In the future, the pathogenicity of new mutations in obesity-related genes identified in our cohort is planned to be confirmed by functional testing *in vitro*. Moreover, the DNA samples from the parents of the patient with a confirmed mutation will be analyzed.

## 6 Data management

The patients’ data will be anonymized and stored in each center (paper CRF). All data from paper CRF will be electronically documented in a password-secured database. An electronic platform will be developed for data storage and statistical analysis. The statistical analysis of the results will be conducted with software dedicated to analyzing medical data.

## 7 Discussion

Nowadays, mutations in at least 50 genes are known to be related to monogenic obesity, and many others are tested (1, 8, 17–19). Many of them are involved in the leptin–proopiomelanocortin pathway. In our project, we are going to examine Polish SEOO children for the presence of mutations in 11 genes, most frequently related to monogenic severe obesity with early onset (**Table 1**) (17, 20, 21). In addition, we performed further analysis of other, less frequent, obesity-related genes (6, 18).

In all patients with those mutations, both hyperphagia, characterized by extreme food-seeking behavior, and early onset of obesity are typical symptoms. In most patients, there are also other diverse symptoms that are characteristic of each mutation (Table 1). Mutations in the *MC4R* are the most common form of inherited SEOO with a prevalence of 0.5%–5.8% in different populations (22). Among the obese Czech child population, similar to the Polish one, the prevalence of *MC4R* homozygous and heterozygous mutations is 2.4% (23). Other mutations are less frequent. It is suggested that leptin deficiency or leptin receptor defects, inherited in an autosomal recessive pattern, could be found even in up to 3% of patients with SEOO (24). So far, 100 patients with SEOO caused by leptin gene mutation were identified. The prevalence of leptin receptor gene (*LEPR*) mutations can reach even 2%–3% in certain populations (24). Mutations in proopiomelanocortin (*POMC*) and proprotein convertase subtilisin/kexin type 1 (*PCSK1*) genes were identified in 10 patients for each gene (8).

The establishment Polish database of children with severe obesity and assessment of bio-active leptin in children with severe obesity were partly met between 2015 and 2019 when the Polish–German consortium was established to implement the “Early-onset Obesity and Leptin—German-Polish Study (EOL-GPS)” project. Fifty SEOO children were recruited from three Polish medical centers (Katowice, Rzeszów, and Szczecin) and one German center (Ulm). Clinical profile, leptin, and biologically active leptin were determined. In this cohort with SEOO, we identified no new cases of children with leptin deficiency or bio-inactive leptin. However, relative leptin deficiency in children with SEOO could be suspected (25). In order to further explore this important topic, the Polish–German consortium decided to continue its cooperation. The current study is the first to focus on monogenic obesity in Polish SEOO children, to establish the prevalence of the most common monogenic lesions as a cause of severe obesity, and to identify new mutations in obesity-related genes specific to the Polish population. In the future, the pathogenicity of new mutations in obesity-related genes identified in our cohort is planned to be confirmed by functional testing *in vitro*.

Based on the rarity of monogenic gene variants as the cause of obesity, the question has to be posed whether there is a justification to search for them. Aside from purely scientific reasons—finding a new, population-specific mutation related to SEOO—the justification is given by the fact that during the past years, mutation-specific treatments have been developed. Patients with leptin deficiency as well as patients with biologically inactive leptin can be treated by administration of recombinant human leptin (metreleptin) (24–27). MC4R agonist, setmelanotide, is now approved for the treatment in patients with POMC, LEPR, and proprotein convertase subtilisin/kexin type 1 (PCSK1) deficiencies (28–31). It is also known that patients with some mutations can be successfully treated with well-known drugs; e.g., glucagon-like peptide 1 (GLP-1) agonist is effective in weight reduction in patients with *MC4R* mutations, and obesity related to Kinase Suppressor of Ras-2 (*KSR2*) mutation is well treated with metformin (21, 28, 30). Identification of monogenic background is also important in patients’ qualification for bariatric surgery. Cooiman et al. (17) showed that patients with *MC4R* mutations achieved superior weight loss after primary Roux-en-Y gastric bypass compared with sleeve gastrectomy.

There are no data regarding the prevalence of severe obesity or monogenic obesity in Polish children. However, looking at the data available for the Czech Republic where 2.2% of children are severely obese, we can estimate that at least the same percentage of Polish children also suffer from severe obesity (32). This figure would be equivalent to an estimated 150,000 Polish children. Most probably because of the genetic diversity of our population and the lack of ethnic minorities with high consanguinity in our country, the prevalence of monogenic obesity is expected to be rather low. If we assume that it is at the level of 3%, it means that about 4,500 children in Poland can be obese due to a mutation in a single gene. In some of them, more than lifestyle intervention and diet can be offered, and specific treatment can be used.

## 8 Study limitation

The study is a cross-sectional analysis. Our sample size is limited; therefore, all cases of monogenic obesity in the Polish population will not be detected. The active recruitment is taking place only in four centers in Poland. Despite the vast informational campaign, there is the risk of the underrepresentation of patients from the regions located at larger distances from the recruiting centers. Moreover, we realized that we are going to analyze a limited group of the selected genes, not covering all possible reasons for monogenic obesity. For this reason, we decided to bank the same genetic material from every patient for future analysis, if it will be possible to perform.

## Ethics statement

The study will be conducted according to the Declaration of Helsinki on “Ethical Principles for Medical Research in Humans” (9 July 2018). The study was approved by the local ethics committees (No. PCN/CBN/0022/KB1/137/I/21/22, KBETUJ 1072.6120.69.2022, KB-006/12/2022). At each participating institution, informed written consent will be obtained from every patient’s parents/guardians and every patient above the age of 13 years.

## Author contributions

AZ, AM, EP, MR, MWa, EM-T, MWo, BF, and SB: conceived the project. AZ, MB-M, and MM: wrote the original draft. MWo, EP, and AM: responsible for conducting the literature review. EM-T and MR: participated in revising the work for important intellectual content. All authors contributed to the article and approved the submitted version.

## Funding

This research was funded in whole by National Science Center, Poland (2021/41/B/NZ5/01676). For the purpose of Open Access, the author has applied a CC-BY public copyright licence to any Author Accepted Manuscript (AAM) version arising from this submission.

## Conflict of interest

Author BF is employed by Mediagnost GmbH.

The remaining authors declare that the research will be conducted in the absence of any commercial or financial relationships that could be construed as a potential conflict of interest.

## Publisher’s note

All claims expressed in this article are solely those of the authors and do not necessarily represent those of their affiliated organizations, or those of the publisher, the editors and the reviewers. Any product that may be evaluated in this article, or claim that may be made by its manufacturer, is not guaranteed or endorsed by the publisher.

## Appendix 1

Medical history of patient and patient’s family.

**Table:**

Patient code	Date
**Date of birth**	
**Birth weight [g]**	
**Birth length [cm]**	
**Head circumference at birth [cm]**	
**Length of pregnancy[weeks]**	
**Apgar score**	
**The age when obesity occurred**	
**Mother’s age**	
**Mother’s height [cm]**	
**Mother’s weight [kg]**	
**Father’s age**	
**Father’s height [cm]**	
**Father’s weight [kg]**

**Table:**

**Sibling’s age**				
**Sibling’s height [cm]**				
**Sibling’s weight [kg]**

**Table:**

**Body weight available from med. data [kg] (date/value)**						
**Length/height available from med. data [cm] (date/value)**						
**Family history**	Obesity	Arterial hypertension	Diabetes mellitus (type 2/ during pregnancy)	Lipids disturbances (hypercholesterolemia/ hypertriglyceridemia)	Cardiovascular diseases (coronary disease, stroke, MI)	Others (endocrine disorders, neurological, suggesting monogenic obesity)
**Who**						

## Appendix 2 Questionnaire regarding the symptoms characteristic for specific mutation.

**Table:**

Patientcode…………………………………………………….		Date………………………………		
	Yes	No	Not known	Comment
Facial dysmorphia				
Somatomegaly				
Frequent, severe infections (immunodeficiency)				
**Endocrine disorders**				
Growth hormone deficiency				
Hypothyroidism				
Adrenal insufficiency				
Hypogonadism				
Diabetes insipidus				
Neurological disorders				
Intellectual disability/ developmental delay				
Behavioral problems – ADHD				
Autism				
Memory problems				
Impaired pain sensation				
Skin (pale) and hair (red) abnormal pigmentation				
Acanthosis nigricans/ hyperinsulinemia				
Hypoglycemic episodes				
Neonatal diarrhoea				

## Appendix 3A

**Table:**

Patient code	Date				
	Never	Rarely	Some-times	Often	Always
My child loves food	□	□	□	□	□
My child eats more when worried	□	□	□	□	□
My child has a big appetite	□	□	□	□	□
My child finishes his/her meal quickly	□	□	□	□	□
My child is interested in food	□	□	□	□	□
My child is always asking for a drink	□	□	□	□	□
My child refuses new foods at first	□	□	□	□	□
My child eats slowly	□	□	□	□	□
My child eats less when angry	□	□	□	□	□
My child enjoys tasting new foods	□	□	□	□	□
My child eats less when s/he is tired	□	□	□	□	□
My child is always asking for food	□	□	□	□	□
My child eats more when annoyed	□	□	□	□	□
If allowed to, my child would eat too much	□	□	□	□	□
My child eats more when anxious	□	□	□	□	□
My child enjoys a wide variety of foods	□	□	□	□	□
My child leaves food on his/her plate at the end of a meal	□	□	□	□	□
My child takes more than 30 minutes to finish a meal	□	□	□	□	□

**Table:**

	**Never**	**Rarely**	**Some-times**	**Often**	**Always**
Given the choice, my child would eat most of the time	□	□	□	□	□
My child looks forward to mealtimes	□	□	□	□	□
My child gets full before his/her meal is finished	□	□	□	□	□
My child enjoys eating	□	□	□	□	□
My child eats more when she is happy	□	□	□	□	□
My child is difficult to please with meals	□	□	□	□	□
My child eats less when upset	□	□	□	□	□
My child gets full up easily	□	□	□	□	□
My child eats more when s/he has nothing else to do	□	□	□	□	□
Even if my child is full up s/he finds room to eat his/her favourite food	□	□	□	□	□
If given the chance, my child would drink continuously throughout the day	□	□	□	□	□
My child cannot eat a meal if s/he has had a snack just before	□	□	□	□	□
If given the chance, my child would always be having a drink	□	□	□	□	□
My child is interested in tasting food s/he hasn’t tasted before	□	□	□	□	□
My child decides that s/he doesn’t like a food, even without tasting it	□	□	□	□	□
If given the chance, my child would always have food in his/her mouth	□	□	□	□	□
My child eats more and more slowly during the course of a meal	□	□	□	□	□

## Appendix 3b

**Table:** Three-Factor Eating Questionnaire (TFEQ).

Patient code	Date			
	Definitely yes	Rather yes	Rather not	Definitely not
I take small portions on purpose, in order to control my body mass				
I eat when I feel nervous				
The company of someone eating makes me so hungry I have to eat too				
I overeat when I feel sad				
When I see something tasty I get so hungry I immediately have to eat				
I often feel so hungry that I could eat endlessly				
I am always hungry, therefore I cannot stop eating until I empty my plate				
Food comforts me when I feel lonely				
I consciously control the amount of my meals not to gain weight				
I abstain from some food because they make me gain weight				
I am always so hungry I can eat anytime				
Can you consciously eat less than you would like to?				
How much do you restrain eating? Check the 1-8 scale (1 - I never restrain eating/ 8 - I always restrain eating)	2. 3. 4. 5. 6. 7. 8			

## Appendix 4

Data from physical examination, biochemical and hormonal tests results.

**Table:**

Patient code	Date of investigation	
Physical examination		
Height [cm]		
Weight [kg]		
Waist circumference [cm]		
Head circumference [cm]		
Blood pressure [mmHg]		
Pubertal stage [Tanner scale]		
Body composition (by bioimpedance) – not obligatory	yes/no (if yes please attach the result)

**Table:**

**Fasting blood results**	**value**	**unit**	
Glucose			
Insulin			
Total cholesterol			
HDL cholesterol			
LDL cholesterol			
Triglycerides			
ALT			
TSH			
FT4			
**Others (esp. US of the abdomen – liver echogenicity, OGTT, Cortisol)**			

## References

[1] AbouHashem N Zaied RE Al-Shafai K Nofal M Syed N Al-Shafai M . The spectrum of genetic variants associated with the development of monogenic obesity in Qatar. Obes Facts. (2022) 15(3):357–65. doi: 10.1159/000521851 PMC9210005 35026759

[2] Kohlsdorf K Nunziata A Funcke JB Brandt S von Schnurbein J Vollbach H . "Frühkindlicher BMI-verlauf bei monogener adipositas". Medizinische Genetik (2017) 29 4:360–4. doi: 10.1007/s11825-017-0167-x"

[3] Kohlsdorf K Nunziata A Funcke JB Brandt S von Schnurbein J Vollbach H . Early childhood BMI trajectories in monogenicobesity due toleptin, leptinreceptor, and melanocortin 4 receptordeficiency. Int J Obes (Lond). (2018) 42(9):1602–9. doi: 10.1038/s41366-018-0049-6 29568105

[4] Skinner AC Ravanbakht SN Skelton JA Perrin EM Armstrong SC . Prevalence of obesity and severe obesity in US children, 1999-2016. Pediatrics (2018) 141:e20173459. doi: 10.1542/peds.2017-3459 29483202 PMC6109602

[5] Cadenas-Sanchez C Intemann T Labayen I Artero EG Alvarez-Bueno C Sanchis-Moysi J . Prevalence of severe/morbid obesity and other weight status and anthropometric reference standards in Spanish preschool children: The PREFIT project. Pediatr Res (2020) 87:501–10. doi: 10.1038/s41390-019-0325-8 30776792

[6] Akinci A Türkkahraman D Tekedereli İ Özer L Evren B Şahin İ . Novel mutations in obesity-related genes in Turkish children with non-syndromic early onset severe obesity: A multicentre study. J Clin Res Pediatr Endocrinol (2019) 11:341–9. doi: 10.4274/jcrpe.galenos.2019.2019.0021 PMC6878344 30991789

[7] De Rosa MC Chesi A McCormack S Zhou J Weaver B McDonald M . Characterization of rare variants in MC4R in African American and Latino children with severe early-onset obesity. J Clin Endocrinol Metab (2019) 104:2961–70. doi: 10.1210/jc.2018-02657 PMC6546308 30811542

[8] Thaker VV . Genetic and epigenetic causes of obesity. Adolesc Med State Art Rev (2017) 28:379–405. doi: 10.1542/9781581109405-genetic 30416642 PMC6226269

[9] Šket R Kotnik P Bizjan BJ Kocen V Mlinarič M Tesovnik T . Heterozygous genetic variants in autosomal recessive genes of the leptin-melanocortin signalling pathway are associated with the development of childhood obesity. Front Endocrinol (Lausanne). (2022) 13:832911. doi: 10.3389/fendo.2022.832911 35574020 PMC9105721

[10] AbouHashem N Al-Shafai K Al-Shafai M . The genetic elucidation of monogenic obesity in the Arab world: a systematic review. J Pediatr Endocrinol Metab (2022) 35(6):699–707. doi: 10.1515/jpem-2021-0710 35437977

[11] Statistic polands (2022). Available at: https://bdl.stat.gov.pl/bdl/dane/podgrup/tablica.

[12] Wardle J Guthrie CA Sanderson S Rapoport L . Development of the children's eating behaviour questionnaire. J Child Psychol Psychiatry (2001) 42:963–70. doi: 10.1111/1469-7610.00792 11693591

[13] Dzielska A Mazur J Małkowska-Szkutnik A Kołoło H . Adaptation of the tree-factor eating questionnaire (TFEQ-13) for school-aged adolescents in a population study. ProblHigEpidemiol (2009) 90:362–9.

[14] Available at: https://cukrzyca.info.pl/aktualnosc/zalecenia-kliniczne-dot-postepowania-u-chorych-z-cukrzyca-2021-stanowisko-ptd.

[15] Wabitsch M Prizdum L Ranke M von Schnurbein J Moss A Brandt S . Measurement of immunofunctional leptin to detect and monitor patients with functional leptin deficiency. Eur J Endocrinol (2017) 176:315–22. doi: 10.1530/EJE-16-0821 PMC5292973 28007844

[16] Blum WF Englaro P Hanitsch S Juul A Hertel NT Müller J . Plasma leptin levels in healthy children and adolescents: dependence on body mass index, body fat mass, gender, pubertal stage, and testosterone. J ClinEndocrinolMetab. (1997) 82(9):2904–10. doi: 10.1210/jcem.82.9.4251 9284717

[17] Cooiman MI Kleinendorst L Aarts EO Janssen IMC van Amstel HKP Blakemore AI . Genetic obesity and bariatric surgery outcome in 1014 patients with morbid obesity. Obes Surg (2020) 30:470–7. doi: 10.1007/s11695-019-04184-w 31650404

[18] Paolacci S Pompucci G Paolini B Del Ciondolo I Miggiano GAD Aquilanti B . Mendelian non-syndromic obesity. Acta BioMed (2019) 90:87–9. doi: 10.23750/abm.v90i10-S.8766 PMC7233639 31577261

[19] Durmaz A Aykut A Atik T Özen S AyyıldızEmecen D Ata A . A new cause of obesity syndrome associated with a mutation in the carboxypeptidase gene detected in three siblings with obesity, intellectual disability and hypogonadotropic hypogonadism. J Clin Res Pediatr Endocrinol (2021) 13(1):52–60. doi: 10.4274/jcrpe.galenos.2020.2020.0101 32936766 PMC7947731

[20] Von Schnurbein J Wabitsch M . Monogene adipositas. pathophysiologie – diagnostik – therapieoptionen. Medizinische Genetik (2017) 4:348–59. doi: 10.1007/s11825-017-0157-z

[21] Pearce LR Atanassova N Banton MC Bottomley B van der Klaauw AA Revelli JP . KSR2 mutations are associated with obesity, insulin resistance, and impaired cellular fuel oxidation. Cell (2013) 155:765–77. doi: 10.1016/j.cell.2013.09.058 PMC3898740 24209692

[22] El-Sayed Moustafa JS Froguel P . From obesity genetics to the future of personalized obesity therapy. Nat Rev Endocrinol (2013) 9:402–13. doi: 10.1038/nrendo.2013.57 23529041

[23] Hainerová I Larsen LH Holst B Finková M Hainer V Lebl J . Melanocortin 4 receptor mutations in obese Czech children: studies of prevalence, phenotype development, weight reduction response, and functional analysis. J Clin Endocrinol Metab (2007) 92:3689–96. doi: 10.1210/jc.2007-0352 17579204

[24] Farooqi IS O’Rahilly S . 20 years of leptin: human disorders of leptin action. J Endocrinol (2014) 223:T63–70. doi: 10.1530/JOE-14-0480 25232148

[25] Zachurzok A Ranke MB Flehmig B Jakubek-Kipa K Marcinkiewicz K Mazur A . Relative leptin deficiency in children with severe early-onset obesity (SEOO) - results of the early-onset obesity and leptin - German-polish study (EOL-GPS). JPediatrEndocrinolMetab (2020) 33:255–63. doi: 10.1515/jpem-2019-0469 31927523

[26] Wabitsch M Funcke JB Lennerz B Kuhnle-Krahl U Lahr G Debatin KM . Biologically inactive leptin and early-onset extreme obesity. N Eng J Med (2015) 372:48–54. doi: 10.1056/NEJMoa1406653 25551525

[27] Wabitsch M Funcke JB von Schnurbein J Denzer F Lahr G Mazen I . Severe early-onset obesity due to bioinactive leptin caused by a p.N103K mutation in the leptin gene. J ClinEndocrinolMetab (2015) 100:3227–30. doi: 10.1210/jc.2015-2263 PMC4570156 26186301

[28] Iepsen EW Zhang J Thomsen HS Hansen EL Hollensted M Madsbad S . Patients with obesity caused by melanocortin-4 receptor mutations can be treated with a glucagon-like peptide-1 receptor agonist. CellMetab (2018) 28:23–32.e3. doi: 10.1016/j.cmet.2018.05.008 29861388

[29] Markham A . Setmelanotide: First approval. Drugs. (2021) 81(3):397–403. doi: 10.1007/s40265-021-01470-9 33638809

[30] Available at: https://www.rhythmtx.com/our-pipeline/.

[31] Poitou C Mosbah H Clément K . MECHANISMS IN ENDOCRINOLOGY: Update on treatments for patients with genetic obesity. Eur J Endocrinol (2020) 183(5):R149–66. doi: 10.1530/EJE-20-0363 33107433

[32] Spinelli A Buoncristiano M Kovacs VA Yngve A Spiroski I Obreja G . Prevalence of severe obesity among primary school children in 21 European countries. Obes Facts (2019) 12:244–58. doi: 10.1159/000500436 PMC6547273 31030201

